# The evaluation of bariatric surgery effect on cardiac structure and function using transthoracic echocardiography: a cohort study

**DOI:** 10.1186/s12893-024-02328-z

**Published:** 2024-01-25

**Authors:** Firoozeh Abtahi, Malek Atashbarg, Mahdi Rahmanian, Nader Moeinvaziri, Mehdi Bazrafshan, Hanieh Bazrafshan, Farzaneh Moammer, Helia Bazroodi, Abdolali Zolghadrasli, Hamed Bazrafshan drissi

**Affiliations:** 1 Cardiovascular research center, Shiraz university of medical science, Shiraz, Iran; 2 Department of Cardiology, School of Medicine, Shiraz University of Medical Science, Shiraz, Iran; 3 Laparascopy research center, Surgery Department, Shiraz university of medical science, Shiraz, Iran; 4 Clinical Neurology Research Center, Shiraz University of Medical Science, Shiraz, Iran; 5 Student research committee, School of Medicine, Shiraz University of Medical Science, Shiraz, Iran; 6 Department of cardiology, Austin Health, Melbourne, VIC Australia

**Keywords:** Morbid obesity, Bariatric surgery, Echocardiography, Left ventricular dysfunction, Ventricular remodeling

## Abstract

**Background:**

Obesity is a pathology and a leading cause of death worldwide. Obesity can harm multiple organs, including the heart. In this study, we aim to investigate the effect of bariatric surgery and following weight loss on cardiac structure and functions using echocardiography parameters in patients with morbid obesity.

**Methods:**

In this cohort study, 30 patients older than 18 with BMI > 40 or BMI > 35 and comorbidity between March 2020 to March 2021 were studied. The patients underwent transthoracic echocardiography before and after six months of the bariatric surgery.

**Results:**

In total, 30 patients (28 women, 93.3%) with a mean age of 38.70 ± 9.19 were studied. Nine (30%) were diabetic, and 9 (30%) had hypertension. After six months of bariatric surgery, all physical measurements, including weight, Body mass index, and Body surface area, decreased significantly (*p* < 0.001). After bariatric surgery, all parameters regarding left ventricular morphology, including left ventricular mass, interventricular septal thickness, left ventricular posterior wall thickness, left ventricular end-systolic diameter, and left ventricular end-diastolic diameter, improved significantly (*p* < 0.001). Also, LVEF rose post-bariatric surgery (*p* < 0.001). TAPSE parameter indicating right ventricular function also improved (*p* < 0.001). Right ventricular diameter, left atrium volume, and mitral inflow E/e^’^ decreased significantly (*p* < 0.001).

**Conclusion:**

Systolic and diastolic parameters refine significantly after bariatric surgery in patients with obesity. Bariatric surgery lead to significant cardiac structure and function improvement.

## Introduction

Obesity is a pathology and a leading cause of death worldwide and is defined as the accumulation of excessive adipose tissue in the body. Obesity is the second most common cause of preventable death after smoking and is a public health burden [1]. The prevalence of obesity has doubled since 1980, and approximately one-third of the global population is affected with obesity [2]. The same prevalence is calculated in Iran as well [3]. As a chronic disease, obesity has multifactorial etiology, including excessive energy intake, a sedentary lifestyle, and different genetic traits interacting with each other [4]. While there is no specific pharmacological treatment for obesity, lifestyle modification is the cornerstone of obesity management. Combining diet, physical activity, and behavior therapy can result in at least 10% weight loss [5]. Pharmacotherapy is recommended for patients whose body mass index (BMI) equals or exceeds 30 and whose lifestyle modification fails [6]. When lifestyle modification and pharmacotherapy fail, bariatric surgery is a therapeutic option in individuals with a BMI more than 40 or BMI more than 35 with comorbidities. Standard bariatric surgeries include Biliopancreatic diversion, sleeve gastrectomy, adjustable gastric banding, and Roux-en-Y gastric bypass [7].

Obesity is an independent risk factor for cardiovascular disorders, including atherosclerosis, hypertension, coronary artery disease, and myocardial infarction [8]. Also, obesity can lead to structural heart remodeling, including ventricular and atrial hypertrophy and cardiomyopathy, ultimately resulting in systolic and diastolic dysfunction [9, 10]. Finally, if obesity remains untreated, it may lead to heart failure, a complex clinical syndrome with high morbidity and mortality [11]. As a result, obesity treatment leads to an individual’s longevity and quality of life and improves public health parameters. In this study, we aimed to investigate the effect of bariatric surgeries on cardiac structure improvement through echocardiographic parameters amongst patients with morbid obesity in a referral hospital in south Iran.

## Methods

### Study design and population

In this cohort study, 43 patients older than 18 with BMI > 40 or BMI > 35 and comorbidity who were referred to the Mother and Child Hospital, Shahid Faghihi Hospital, and Hafez Hospital affiliated with Shiraz University of Medical Sciences, Shiraz, Iran, between March 2020 to March 2021, were selected. The exclusion criteria in this study were prior history of any cardiac surgery or intervention, left ventricular ejection fraction less than 50%, valvular heart disease, prior history of coronary artery disease, heart rhythm other than sinus, and patients with poor echocardiography view. Based on exclusion criteria, 13 patients were omitted from the study. The remaining 30 patients underwent transthoracic echocardiography (TTE) before and after six months of the bariatric surgery. We assumed that significant weight loss occurs six months after bariatric surgery [12]. Patient’s demographic data, including age, gender, prior medical disease, weight (Kg), height (m^2^), body mass index (BMI) (Kg/m^2^), Waist to hip ratio (WHR), and body surface area (BSA) (m^2^) were also recorded. This study was approved by the Ethics Committee of the Shiraz University of Medical Sciences (IR.SUMS.MED.REC.1401.421).

### Echocardiographic measurements

Transthoracic echocardiography was performed in included patients at baseline right before bariatric surgery and six months after surgery using Vivid E9 Ultrasound Echo Machine. Each parameter was measured three times, and the average was reported as the final result. The patients were examined in lateral left decubitus position, and cardiac dimensions and functions were measured according to current recommendations [13]. Left ventricular (LV) dimensions in the end systole (LVESD) and diastole (LVEDD) were measured. Septum (ST) and posterior wall thickness (PWT) were also measured at the end diastole. LV mass was calculated using the formula: LVM = 0.8 [1.04 (IVSTd + LVIDD + PWTd) ^3^ − (LVIDd)^3^] + 0.6 g. LV mass index was measured by dividing LV mass to body surface area. LV end-systolic diameter (LVESD) and LV end-diastolic diameter (LVEDD). Changes in systolic function were evaluated by measuring left ventricular ejection fraction (LVEF) using the modified Simpson’s method: [(LV end-diastolic volume) – (LV end-systolic volume)]/(LV end-diastolic volume). LV end-systolic volume (LVESV) and LV end-diastolic volume (LVEDV) were also measured. Right ventricular (RV) dimensions were calculated in base and mid-level end diastole. Left atrium (LA) diameter and volume were also measured in the end-systole. LA volume index was measured by dividing LA volume to body surface area.

Early to late diastolic transmitral flow velocity (E/A) was used to assess diastolic function, and E to early diastolic mitral annular tissue velocity (E/e’) to estimate LV filling pressures. Alterations in diastolic function were assessed by pulsed-wave tissue Doppler recordings of peak early (E-wave) and the lateral portion of the mitral annulus to obtain the early diastolic e′-wave velocity. Septal e’ was measured and averaged with lateral e’ to obtain the final e’ value. The mitral inflow E velocity to tissue Doppler e′ (E/e′) ratio was used as an index of LV filling. Right ventricle function was determined by Tricuspid annular plane systolic excursion (TAPSE).

Surgical technique.

The same surgical team performed either laparoscopic Roux-en-Y gastric (LRYGB) bypass or laparoscopic sleeve gastrectomy (LSG) on the patients. In those patients with BMI between 35 and 40 Kg/m^2^, as a first-step treatment in cases with BMI more than 50 Kg/m^2^, and when drug malabsorption was to be avoided LSG was done. The other patients in whom faster weight reduction rate was indicated underwent LRYGB [14, 15]. In LSG a longitudinal resection of the stomach from His angle to approximately 5 cm proximal to the pylorus was done. In LRYGB an approximately 120-cm ante colic Roux limb with 25-mm pouch-jejunostomy was performed.

Statistics.

Kolmogorov smirnov test was used to assess data normality. Descriptive data were presented as mean ± standard deviation (SD), frequency, and percentage. Paired-Samples T-test was used for bivariate analysis. A p-value (p) of less than 0.05 was considered statistically significant. R version 4.2.3 was used for data analysis.

## Results

In total, 30 patients (28 women, 93.3%) with a mean age of 38.70 ± 9.19 were studied. Nine (30%) were diabetic, and 9 (30%) had hypertension (Table 1).

**Table 1:** Basic characteristics of patients

Age (Mean ± SD)	38.70 ± 9.19
Gender: Female (N,%)	28 (93.3%)
Diabetes Mellitus ( N,%)	9 (30%)
Hypertension ( N,%)	9 (30%)
Hyperlipidemia ( N,%)	10 (33.3%)
Systolic blood pressure (Mean ± SD)	123.66 ± 9.99
Diastolic blood pressure (Mean ± SD)	78.33 ± 8.44
Height (Mean ± SD) cm	163.3 ± 8.5

After six months of bariatric surgery, all physical measurements decreased, including weight, BMI, BSA, and waist-to-hip ratio. (Table 2).

**Table 2:** Mean patients’ physical measurements before and after six months of surgery

Item	Before surgery	After surgery	p-Value^*^
Weight (Kg)	113.37 ± 15.61	83.75 ± 12.38	< 0.001
BMI	42.38 ± 5.46	31.38 ± 3.74	< 0.001
BSA (m^2^)	2.28 ± 0.21	1.94 ± 0.17	< 0.001
Waist-to-hip ratio	1.05 ± 0.03	0.96 ± 0.04	< 0.001

BMI = Body mass index, BSA = Body surface area

*Paired-Samples T-test

After bariatric surgery, all parameters regarding left ventricular morphology, including LV mass, IVST, LVPWT, LVESD, and LVEDD decreased significantly. LVEF improved post-bariatric surgery. TAPSE parameter indicating right ventricular function improved as well. Right ventricular diameter, left atrium volume and mitral inflow E/e^’^ decreased significantly (Table 3).

**Table 3:** Changes in echocardiographic parameters after bariatric surgery

Item	Before surgery	After surgery	p-Value^*^
Left ventricular morphology			
LV mass (g)	177.19 ± 60.35	143.71 ± 37.74	< 0.001
LV mass index (g/m^2^)	80.58 ± 29.38	74.49 ± 21.14	0.009
IVST (mm)	11.23 ± 2.47	10.01 ± 1.63	< 0.001
LVPWT (mm)	9.6 ± 1.56	8.81 ± 1.24	< 0.001
LVESD (mm)	33.76 ± 2.88	31.26 ± 2.86	< 0.001
LVEDD (mm)	46.46 ± 4.09	44.06 ± 3.21	< 0.001
Left ventricular systolic function			
LVEF (%)	57.70 ± 2.50	59.83 ± 0.91	< 0.001
LVEDV (ml)	106.28 ± 28.32	90.33 ± 19.46	< 0.001
LVESV (ml)	42.52 ± 10.39	34.45 ± 8.16	< 0.001
Left ventricular diastolic function			
e’ lateral, cm/s	11.20 ± 2.82	12.50 ± 2.31	< 0.001
e’ septal, cm/s	8.06 ± 2.31	9.16 ± 1.64	< 0.001
E/e’	8.45 ± 2.07	7.43 ± 1.44	< 0.001
E/A	0.99 ± 0.15	1.0 ± 0.13	0.089
Right ventricular morphology			
Base RVD (mm)	38.03 ± 6.77	36.05 ± 2.88	< 0.001
Mid RVD (mm)	29.23 ± 4.34	28.13 ± 3.83	< 0.001
Right ventricular function			
TAPSE, mm	20.60 ± 1.73	21.86 ± 1.45	< 0.001
Left atrium morphology			
LA diameter, mm	35.72 ± 4.53	34.74 ± 3.55	< 0.001
LA volume, ml	52.55 ± 14.30	47.0 ± 13.40	< 0.001
LA volume index, ml/m^2^	23.35 ± 6.46	24.41 ± 7.40	0.019

LVEF: left ventricular ejection fraction, LV mass: left ventricular mass, LVEDD: left ventricular end-diastolic diameter, LVEDV: left ventricular end-diastolic volume, LVESD: left ventricular end-systolic diameter, LVESV: left ventricular end-systolic volume, LVPWT: left ventricular posterior wall thickness, IVST: interventricular septal thickness, RVD: right ventricular diameter, LA: left atrium, TAPSE: Tricuspid annular plane systolic excursion

*Paired-Samples T-test

## Discussion

In this study, we aimed to investigate the effects of bariatric surgery on heart structure and function. We found that bariatric surgery significantly reduced patients’ physical parameters, including weight and BMI subsequently. Other measures, including BSA and WHR, also reduced. These results concord with the prior studies, which confirmed the effect of bariatric surgery on treating obesity [16, 17].

After a six-month follow-up, almost all parameters regarding cardiac structure and functions were improved significantly in this study. In previous studies it was shown that weight loss can enhance cardiac structure function. In a study done by karwi et al., weight loss improved cardiac function by increasing ejection fraction and decreased ventricular hypertrophy [18]. Another study done by Zhao et al. showed that weight loss is associated with decreased cardiovascular related mortality [19].

Regarding cardiac structure and function alterations after bariatric surgery, some studies were in line with ours, and some were in contrast. The differences may be related to the variety in the severity of obesity and its adverse effect on heart structure and function at baseline, different sample sizes and follow-up duration, or differences in the study population regarding genetics and ethnicity.

LVEF, which is an indicator of LV function increased. Some prior studies were in line with this result. Vest et al. studied 48 patients with LVEF lower than 50% who underwent bariatric surgery. They reported significant mean pre- to post-operative LVEF improvement [20]. Also, a meta-analysis study by Gherbesi et al. showed that bariatric surgery improves LVEF [21]. However, Oliveras et al., who studied 62 patients with obesity and followed them for a year post-bariatric surgery, found no significant increase in LVEF [22]. In their study, Solagnioni et al. used the radiological and non-radiological Haller index to show that impaired LV structure and function in patients with obesity may be related to extrinsic abdominal and thoracic compression rather than intrinsic dysfunction [23].

LV mass and LVPWT were also reduced following weight loss in this study. In their review article, Graspa et al. confirmed this result [24].

We also found that LVESV and LVEDV decreased after six months’ post-bariatric surgery. These results were in line with the study done by Henry et al., who performed cardiac magnetic resonance on 62 patients with obesity before and after bariatric surgery [25].

TAPSE, a clinically helpful echocardiographic measure of global right ventricle function, increased after six months. Similar to this result, based on a two-year follow-up of 423 patients with obesity who underwent gastric bypass surgery, right ventricular function improved [26]. in contrast to this result, Oliver et al. found no significant alteration in TAPSE post-bariatric surgery [22].

RV diameter decreased significantly in our patients. Similarly, Garza et al. studied 57 patients with obesity who underwent gastric bypass, followed them for six months, and reported improved RV function following weight loss [27].

We found that following bariatric surgery, LA volume and diameter decrease significantly. These results were similar to previous studies [28, 29]. However, Sorimachi et al., reported that LA volume and estimated LA pressures increased after bariatric surgery and weight loss [30]. In our study LA volume index increased after bariatric surgery. This is due to more significant reduction in body surface area than LA volume after bariatric surgery.

In this study, the mitral inflow E/A ratio, which indicates the diastolic function, did not increase significantly; however, mitral inflow E/e^’^ decreased significantly following weight loss in our patients, showing a reduction in left ventricular filling pressure and improvement in diastolic function. Previous studies confirmed this result as well [24, 31].

Considering the results of this study and previous researches, bariatric surgery can significantly improve cardiac structure and function. Although these effects have been associated with weight loss, since no study has proved the causality of weight loss and cardiac structure and function improvements, we can not claim that weight loss is the only reason for these effects. Further studies including more variables regarding lifestyle modification expected to happen to patients undergoing bariatric surgery, such as changes in dietary patterns, exercise, etc., and chest wall conformation, are essential.

### Limitations

The notable strength of our study was its cohort design and its limitation was a small number of patients which was due to COVID-19 pandemic that led to significant reduction in elective surgeries. Also, due to patients’ obesity at baseline, we could not perform strain echocardiography for evaluation of cardiac function. This study did not measure patients’ chest wall conformation before and after bariatric surgery.

## Conclusion

In conclusion, systolic and diastolic parameters obtained by transthoracic echocardiography refined significantly after bariatric surgery in people with obesity. As a result, bariatric surgery led to significant cardiac structure and function improvement.

## Data Availability

Data of the participants can be requested from the authors. Please write to the corresponding author if you are interested in such data.

## References

[1] Panuganti KK, Nguyen M, Kshirsagar RK. Obesity. [Updated 2022 Aug 8]. In: StatPearls [Internet]. Treasure Island (FL): StatPearls Publishing; 2023 Jan-. Available from: https://www.ncbi.nlm.nih.gov/books/NBK459357/.

[2] Ataey A, Jafarvand E, Adham D, Moradi-Asl E (2020). The relationship between obesity, overweight, and the Human Development Index in World Health Organization Eastern Mediterranean Region Countries. J Prev Med Public Health.

[3] Akbari-Khezrabadi A, Zibaeenezhad MJ, Shojaeefard E, Naseri A, Mousavi S, Sarejloo S et al. Can anthropometric indices predict the chance of hypertension? A multicentre cross-sectional study in Iran. BMJ open [Internet]. 2022 2022/11//; 12(11):[e062328 p.]. Available from: http://europepmc.org/abstract/MED/36418117. 10.1136/bmjopen-2022-062328. https://europepmc.org/articles/PMC9685002. https://europepmc.org/articles/PMC9685002?pdf=render. 10.1136/bmjopen-2022-062328 PMC9685002 36418117

[4] Serra-Majem L, Bautista-Castaño I (2013). Etiology of obesity: two key issues and other emerging factors. Nutr Hosp.

[5] Guidelines. (2013) for managing overweight and obesity in adults. Preface to the Expert Panel Report (comprehensive version which includes systematic evidence review, evidence statements, and recommendations). Obesity (Silver Spring). 2014;22 Suppl 2:S40. 10.1002/oby.20822 24961824

[6] Telles S, Gangadhar BN, Chandwani KD (2016). Lifestyle modification in the Prevention and management of obesity. J Obes.

[7] Lin X, Li H, Obesity (2021). Epidemiology, pathophysiology, and therapeutics. Front Endocrinol (Lausanne).

[8] Cercato C, Fonseca FA (2019). Cardiovascular risk and obesity. Diabetol Metab Syndr.

[9] Rider OJ, Lewis AJ, Neubauer S (2014). Structural and metabolic effects of obesity on the myocardium and the Aorta. Obes Facts.

[10] Litwin SE, Adams TD, Davidson LE, McKinlay R, Simper SC, Ranson L, Hunt SC (2020). Longitudinal changes in Cardiac structure and function in severe obesity: 11-Year Follow-Up in the Utah obesity study. J Am Heart Assoc.

[11] Hajouli S, Ludhwani D. Heart Failure And Ejection Fraction. [Updated 2022 Dec 23]. In: StatPearls [Internet]. Treasure Island (FL): StatPearls Publishing; 2023 Jan-. Available from: https://www.ncbi.nlm.nih.gov/books/NBK553115/. 31971755

[12] Nedeljkovic-Arsenovic O, Banovic M, Radenkovic D, Rancic N, Polovina S, Micic D, Nedeljkovic I (2019). The amount of weight loss six months after bariatric surgery: it makes a difference. Obes Facts.

[13] Recommendations for Cardiac Chamber Quantification by Echocardiography in Adults (2016). An update from the American Society of Echocardiography and the European Association of, Cardiovascular Imaging. Eur Heart J Cardiovasc Imaging.

[14] Tucker ON, Szomstein S, Rosenthal RJ (2008). Indications for sleeve gastrectomy as a primary procedure for weight loss in the morbidly obese. J Gastrointest Surg.

[15] Douglas IJ, Bhaskaran K, Batterham RL, Smeeth L (2015). Bariatric surgery in the United Kingdom: a cohort study of weight loss and clinical outcomes in Routine Clinical Care. PLoS Med.

[16] Wolfe BM, Kvach E, Eckel RH (2016). Treatment of obesity: weight loss and bariatric surgery. Circ Res.

[17] Arterburn DE, Telem DA, Kushner RF, Courcoulas AP (2020). Benefits and risks of bariatric surgery in adults: a review. JAMA.

[18] Karwi QG, Zhang L, Altamimi TR, Wagg CS, Patel V, Uddin GM (2019). Weight loss enhances cardiac energy metabolism and function in heart failure associated with obesity. Diabetes Obes Metab.

[19] Zhao Y, Yu BY, Liu Y, Tong T, Liu Y (2018). Weight reduction and cardiovascular benefits: protocol for a systematic review and meta-analysis. Med (Baltim).

[20] Vest AR, Patel P, Schauer PR, Satava ME, Cavalcante JL, Brethauer S, Young JB (2016). Clinical and echocardiographic outcomes after bariatric surgery in obese patients with left ventricular systolic dysfunction. Circ Heart Fail.

[21] Gherbesi E, Cuspidi C, Faggiano A, Sala C, Carugo S, Tadic M. Bariatric surgery and myocardial mechanics: a Meta-analysis of speckle tracking echocardiographic studies. J Clin Med. 2022;11(16). 10.3390/jcm11164655 PMC9410478 36012899

[22] Oliveras A, Molina L, Goday A, Sans L, Riera M, Vazquez S (2021). Effect of bariatric surgery on cardiac structure and function in obese patients: role of the renin-angiotensin system. J Clin Hypertens (Greenwich).

[23] Sonaglioni A, Nicolosi GL, Trevisan R, Granato A, Zompatori M, Lombardo M (2022). Modified Haller index validation and correlation with left ventricular strain in a cohort of subjects with obesity and without overt heart disease. Intern Emerg Med.

[24] Grapsa J, Tan TC, Paschou SA, Kalogeropoulos AS, Shimony A, Kaier T (2013). The effect of bariatric surgery on echocardiographic indices: a review of the literature. Eur J Clin Invest.

[25] Henry JA, Abdesselam I, Deal O, Lewis AJ, Rayner J, Bernard M (2023). Changes in epicardial and visceral adipose tissue depots following bariatric surgery and their effect on cardiac geometry. Front Endocrinol (Lausanne).

[26] Owan T, Avelar E, Morley K, Jiji R, Hall N, Krezowski J (2011). Favorable changes in cardiac geometry and function following gastric bypass surgery: 2-year follow-up in the Utah obesity study. J Am Coll Cardiol.

[27] Garza CA, Pellikka PA, Somers VK, Sarr MG, Collazo-Clavell ML, Korenfeld Y, Lopez-Jimenez F (2010). Structural and functional changes in left and right ventricles after major weight loss following bariatric surgery for morbid obesity. Am J Cardiol.

[28] Garza CA, Pellikka PA, Somers VK, Sarr MG, Seward JB, Collazo-Clavell ML (2008). Major weight loss prevents long-term left atrial enlargement in patients with morbid and extreme obesity. Eur J Echocardiogr.

[29] Aggarwal R, Harling L, Efthimiou E, Darzi A, Athanasiou T, Ashrafian H (2016). The effects of bariatric surgery on Cardiac structure and function: a systematic review of Cardiac Imaging outcomes. Obes Surg.

[30] Sorimachi H, Obokata M, Omote K, Reddy YNV, Takahashi N, Koepp KE (2022). Long-term changes in Cardiac structure and function following bariatric surgery. J Am Coll Cardiol.

[31] Kurnicka K, Domienik-Karłowicz J, Lichodziejewska B, Bielecki M, Kozłowska M, Goliszek S (2018). Improvement of left ventricular diastolic function and left heart morphology in young women with morbid obesity six months after bariatric surgery. Cardiol J.

